# Constitutive Modeling of Time-Dependent Response of Human Plantar Aponeurosis

**DOI:** 10.1155/2014/530242

**Published:** 2014-02-20

**Authors:** P. G. Pavan, P. Pachera, C. Stecco, A. N. Natali

**Affiliations:** ^1^Department of Industrial Engineering, University of Padova, Via G. Marzolo 8, 35131 Padova, Italy; ^2^Centre of Mechanics of Biological Materials, University of Padova, Via G. Marzolo 9, 35131 Padova, Italy; ^3^Department of Molecular Medicine, University of Padova, Via A. Gabelli 63, 35131 Padova, Italy

## Abstract

The attention is focused on the viscoelastic behavior of human plantar aponeurosis tissue. At this purpose, stress relaxation tests were developed on samples taken from the plantar aponeurosis of frozen adult donors with age ranging from 67 to 78 years, imposing three levels of strain in the physiological range (4%, 6%, and 8%) and observing stress decay for 240 s. A viscohyperelastic fiber-reinforced constitutive model with transverse isotropy was assumed to describe the time-dependent behavior of the aponeurotic tissue. This model is consistent with the structural conformation of the tissue where collagen fibers are mainly aligned with the proximal-distal direction. Constitutive model fitting to experimental data was made by implementing a stochastic-deterministic procedure. The stress relaxation was found close to 40%, independently of the level of strain applied. The agreement between experimental data and numerical results confirms the suitability of the constitutive model to describe the viscoelastic behaviour of the plantar aponeurosis.

## 1. Introduction

The plantar aponeurosis is deeply involved in the biomechanical behavior of the foot, being a structural element that directly affects the deformation of the foot arch. The plantar aponeurosis allows the transmission of the forces in the proximal-distal direction of the foot, contributing to keep the medial longitudinal arch conformation [1, 2]. It can be subjected to different pathologies of overuse [3] and systemic origin [4] that can compromise its mechanical functionality. A deeper knowledge about the mechanical properties of constituent materials and about the structural response of the plantar aponeurosis could lead to a better understanding of the biomechanical behavior of the foot, both in physiological and pathological conditions.

Presently, there is few work in the literature about the biomechanics of the plantar aponeurosis. Wright and Rennels [5] investigated plantar aponeurosis elastic properties testing samples taken from adult donors. They found that the plantar aponeurosis shows a nonlinear behavior, with a typical toe region characterized by low stiffness and followed by a strain-stiffening region. The authors referred to a limit of the elastic range at nominal strains of about 6-7%, even if they attested the presence of failure phenomena induced by the setup of the testing apparatus. In the same work, the deformation of the longitudinal arch was evaluated *in vitro* under application of vertical loads on the tibia, estimating the lengthening of the arch for a load up to 900 N and deducing the elongation of the plantar aponeurosis. Gefen [6] estimated by *in vivo* tests a larger compliance of the plantar aponeurosis, attesting a lengthening up to 11-12% strain under a load of 900 N. This larger deformability is in agreement with the outcomes of other pieces of work appeared in the literature [7]. The nonlinear behavior found by Wright and Rennels [5] was experimentally confirmed also by Kitaoka et al. [8].

In the work investigating foot biomechanics by using numerical approaches the plantar aponeurosis is mostly considered as elastic material. The effect of a variation of the plantar aponeurosis conformation on the longitudinal arch deformation and the consequences on the foot structure were investigated by different numerical analyses that assume isotropic linear elastic models [9–11], while an isotropic nonlinear response was considered by Gefen [6] and Cheung et al. [11]. A nonlinear hyperelastic model but with transverse isotropy was considered by Natali et al. [12, 13] to investigate the role of the plantar aponeurosis in distributing plantar pressure.

The present study investigates the stress relaxation of the plantar aponeurosis with the aim of contributing to define a constitutive model capable of representing the time-dependent behaviour. Specific stress relaxation tests were developed on samples taken from three adult donors. The strain range considered was planned on the basis of the outcomes of a previous investigation [14] and taking into account the literature remarks regarding the range of physiological strain [6]. A user-defined fitting procedure [15] was adopted to fit the constitutive model to experimental data. The constitutive model proposed in this work represents a generalization of a fiber-reinforced hyperelastic model of the plantar aponeurosis [13].

## 2. Materials and Methods

### 2.1. Dissection and Treatment of Samples

A total of 24 samples of tissue were taken from the plantar aponeurosis of three male adult donors (age of 67, 67, and 78 years). After the dissection, the samples were frozen and kept at a temperature of −20°C until about 20 minutes before the experimental tests, when they were hydrated with a saline solution (PBS, phosphate buffered solution) at a temperature of 20°C [16]. The saline solution was used to speed up the unfrozen process and to avoid dehydration until the time of testing.

**Figure 1:**
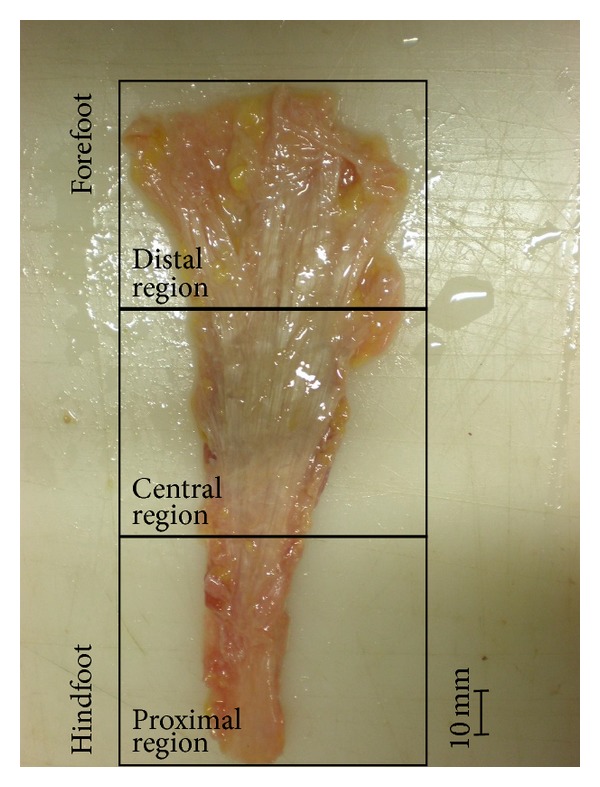
Proximal, central, and distal regions of the plantar aponeurosis.

During the anatomical dissection, the structural conformation of the plantar aponeurosis tissue makes it possible to observe bundles of collagen fibers reinforcing the tissue mainly along the proximal-distal direction. This is in agreement with the fact that, because of its biomechanical function, the plantar aponeurosis is largely loaded in the proximal-distal direction during the loading of the longitudinal foot arch. Single rectangular strips aligned with the proximal-distal direction and with mean width of 5 mm were, therefore, taken from all the three regions composing the plantar aponeurosis: proximal, central, and distal. Typical sample of a plantar aponeurosis is shown in Figure 1. From the three donors were taken 7, 8, and 9 samples, respectively. The dimensions of each sample were evaluated by means of a digital camera and with a subsequent image processing to define thickness and width. The average thickness of the samples was similar for the three donors, with values of 2.4 mm, 2.41 mm, and 2.57 mm, respectively. The samples had a mean total length of 30 mm.

### 2.2. Mechanical Testing

Mechanical tests were carried out by using the Planar Biaxial TestBench Test Instrument Bose Electro-Force (USA) under displacement control with precision of ±0.001 mm and adopting a load cell with precision of ±0.2 N. In order to avoid slipping, two patches of Velcro were interposed between grip surfaces and samples [17]. The gauge length of the samples was 10 mm, corresponding to a mean aspect ratio (length/width) of about 2. Nominal strain values were calculated dividing the current sample length by its initial length value, according to the procedure usually adopted in the literature [5, 18, 19]. Nominal stress was evaluated as the ratio between recorded force and value of undeformed cross-sectional area. The relationship between nominal stress and the measure of stress adopted in the constitutive formulation was obtained exploiting the volume conservation constraint. This assumption is commonly justified by the plentiful presence of water in the tissue [20].

Before stress relaxation tests, all the samples were preconditioned by applying 10-sine shaped strain cycles in the range of 0–2% at frequency of 1 Hz [21]. Three relaxation tests were applied consecutively on each sample, imposing an elongation corresponding to nominal strain of 4%, 6%, and 8%, respectively, and with a rest period between consecutive tests. This rest period was introduced in order to allow for the total development of viscous phenomena in the unloading phase. The elongation ramp before the stress relaxation phase was applied at strain rate of 120%/s in order to limit any viscous process in the tissue, as explained in the discussion section. The stress relaxation process was then monitored for 240 s. The tests were performed in hydrated conditions maintained by pipetting with physiological solution every 30 seconds [22, 23].

### 2.3. Statistical Analysis

The possible effects of strain on the stress relaxation [19] were considered by performing a one-way analysis of variance (ANOVA) (Statgraphics Centurion), comparing stress decay for the three strains imposed, at different time instants of the relaxation process (0.1 s, 6 s, 72 s, and 240 s). These instants were chosen within time intervals that are characteristic of the viscous phenomena investigated. In fact, the stress relaxation processes typically show relaxation time values that span three orders of magnitude starting from the tenth of a second. Further, the additional time instant at the end of the test was included. The minimum significant difference of 0.05 was considered.

### 2.4. Constitutive Model

From anatomical and histological observations, the plantar aponeurosis tissue [24] appears to be made up of a ground matrix and reinforced by collagen fibers that are mainly oriented along the proximal-distal direction. According to the collagen fibers organization, a transversally isotropic scheme was therefore adopted. The viscoelastic response of the tissue was described by assuming a constitutive model in agreement with those proposed for other soft biological tissues [20, 25, 26]. The kinematics is treated following general approach for almost incompressible materials at large strains. The strain measure is defined according to the right Cauchy-Green strain tensor **C** and its volume-preserving part $\tilde{C}$ [27]:

(1) $$\begin{array}{c} C=F^{T}F,\;\;\tilde{C}=J^{-2/3}C, \end{array}$$

where **F** is the deformation gradient and *J* = det⁡**F** is the jacobian of the deformation. In the formulation adopted, the following invariants are considered:

(2) $$\begin{array}{c} {\tilde{I}}_{1}=\mathrm{tr}\left(\tilde{C}\right),\;\;{\tilde{I}}_{4}=\tilde{C}:\left(M⊗M\right), \end{array}$$

where **M** is the unit vector giving the local orientation of the collagen fibers in the undeformed mechanical state. In the specific case considered, it is oriented along the proximal-distal direction according to the above-mentioned anatomical and histological conformation.

The elastic stress response of the tissue is given by defining the following strain energy function:

(3) $$\begin{array}{c} W=U_{\text{mat}}\left(J\right)+{\tilde{W}}_{\text{mat}}\left({\tilde{I}}_{1}\right)+{\tilde{W}}_{\text{fib}}\left({\tilde{I}}_{4}\right), \end{array}$$

where *U*_mat_ and ${\tilde{W}}_{\text{mat}}$ are terms of the ground matrix related to volumetric and volume-preserving strain, respectively, and ${\tilde{W}}_{\text{fib}}$ is a term related to the reinforcing collagen fibers. For the specific tissue under investigation, the strain energy function terms are assumed as follows [13]:

(4) $$\begin{array}{c} U_{\text{mat}}=p\left(J-1\right)\\ {\tilde{W}}_{\text{mat}}=\frac{\mu }{2}\left({\tilde{I}}_{1}-3\right)\\ {\tilde{W}}_{\text{fib}}=\frac{k}{2\alpha }\left[\exp\left(\alpha \langle {\tilde{I}}_{4}-1\rangle -\langle {\tilde{I}}_{4}-1\rangle -1\right)\right], \end{array}$$

where *p* is a stress-like variable that can be interpreted as the hydrostatic pressure, *μ* is a stress-like parameter representing the shear stiffness of the ground matrix at small strains, and *k* (stress-like) and *α* (dimensionless) are parameters related to the stress-strain response of the collagen fibers. The use of the ramp function 〈*x*〉 = (*x* + |*x*|)/2 allows for considering the stiffness contribution of the collagen fibers only in the case of tension, that is, for ${\tilde{I}}_{4}>1$.

The time-dependent response of the tissue is given in terms of the second Piola-Kirchhoff stress tensor by

(5) $$\begin{array}{c} S=2\frac{\partial W}{\partial C}-\sum_{i=1}^{m}Q_{i}, \end{array}$$

where the first term represents the instantaneous stress response at the current time, while **Q**_*i*_ are terms depending on the loading history of the tissue and are related to the developing of viscous phenomena. The evolution over time of **Q**_*i*_ is described by differential equations of the type:

(6) $$\begin{array}{c} {\dot{Q}}_{i}+\frac{1}{\tau_{i}}Q_{i}=\frac{\gamma_{i}}{\tau_{i}}J^{-2/3}\text{DEV}\left[2\frac{\partial W}{\partial C}\right]\;\left(i=1,\ldots ,m\right);\\ \sum_{i=1}^{m}\gamma_{i}\leq 1,\;\tau_{i}>0\\ \underset{t\to -\infty }{\lim}Q_{i}=0, \end{array}$$

where *γ*_*i*_ is the relative stiffness of each viscous process, *τ*_*i*_ is the associated relaxation time, and ${\dot{Q}}_{i}$ are the derivatives with respect to time. The solution of the previous differential equations can be obtained in terms of convolution integrals:

(7) Qi=γiτi∫−∞texp⁡(−(t−s)τi)J−2/3DEV[2∂W∂C]ds(i=1,…,m).

The previous integrals are subjected to simplifications considering the constant level of strain, therefore of the elastic deviatoric stress, during the relaxation process [27].

### 2.5. Constitutive Model Fitting

Aspect ratio of samples and imposed boundary conditions during the tests allows approximating the stress state on the tissue as uniaxial. Having *λ* the stretch in the direction of elongation, transversal stretches are deduced considering the kinematics constraint of incompressibility and assuming a symmetric behavior in the perpendicular directions to that of elongation, recalling the specific alignment of collagen fibers. The value of the elastic stress component is deduced imposing null stress in the perpendicular directions and deducing the related values of viscous stresses **Q**_*i*_ through (7). Because of the level of strain rate (120% s^−1^) applied in the ramp preceding the relaxation process, viscous phenomena in this phase are neglected. The stress decay in the relaxation process is evaluated defining a stress relaxation ratio as a function of time *R*(*t*) = *S*(*t*)/*S*(0), representing the stress at current time normalized to the stress at the initial instant of the relaxation process.

Evaluating the corresponding experimental values of the stress relaxation ratio *R* ^exp⁡^(*t*), it is possible to define a scalar function *χ* estimating the error between numerical results and experimental data:

(8) $$\begin{array}{c} \chi ={\left\{\frac{1}{N}\sum_{i=1}^{N}{\left[1-\frac{R^{\exp}\left(t_{i}\right)}{R\left(\eta ,t_{i}\right)}\right]}^{2}\right\}}^{1/2}, \end{array}$$

where *t*_*i*_ are the *N* time instants of evaluation of the relaxation ratio and *η* is a vector containing the set of the constitutive parameters *γ*_*i*_ and *τ*_*i*_. The optimum set of the constitutive parameters, *η*_opt_, is found by minimizing the error function (8) through the use of a stochastic-deterministic optimization procedure [15, 28].

In order to choose the number of the viscoelastic branches, different fitting procedures were made evaluating the performance of models characterized by one, two, three, and four viscoelastic branches. The growth of the number of the viscous processes leads, as general rule, to a better data fitting but increases also the complexity of the model. In this phase the constitutive model was fitted on experimental data obtained from a limited number of stress relaxation tests.

## 3. Results

**Figure 2:**
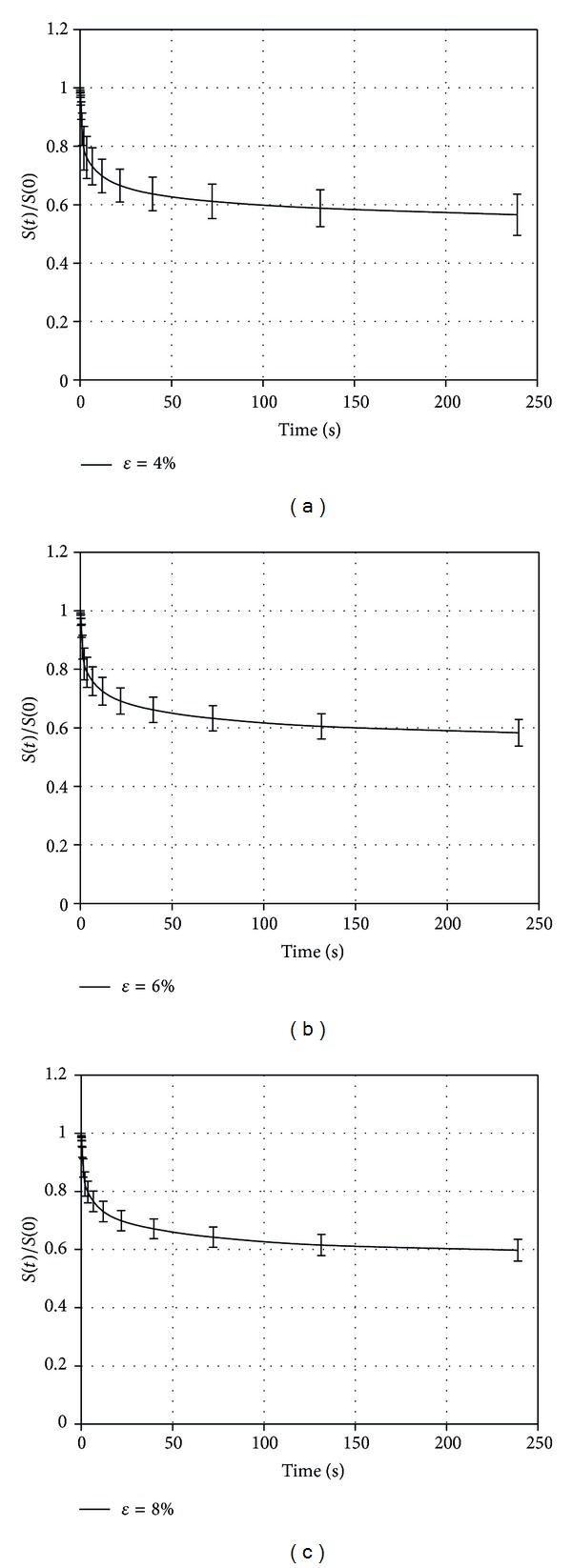
Comparison of average stress relaxation values and SD of the different samples tested at the strain level of 4% (a), 6% (b), and 8% (c), respectively.

Experimental data from stress relaxation tests are reported in terms of normalized stress, expressed by the stress ratio *R* ^exp⁡^(*t*) = *S* ^exp⁡^(*t*)/*S* ^exp⁡^(0) versus time. This representation allows for the proper comparison among experimental outcomes, evaluating the possible dependency of stress relaxation on the imposed level of strain.  Figure 2 shows data observed from stress relaxation tests related to the three values of strain applied.

In Table 1 average values of normalized stress and related SD at the time instants of 0.1 s, 6 s, 72 s, and 240 s for the different experimental tests at 4%, 6%, and 8% of strain applied are reported. Comparing groups characterized by different strain levels, the ANOVA did not show any statistically significant difference for the stress relaxation at any time considered.

**Figure 3:**
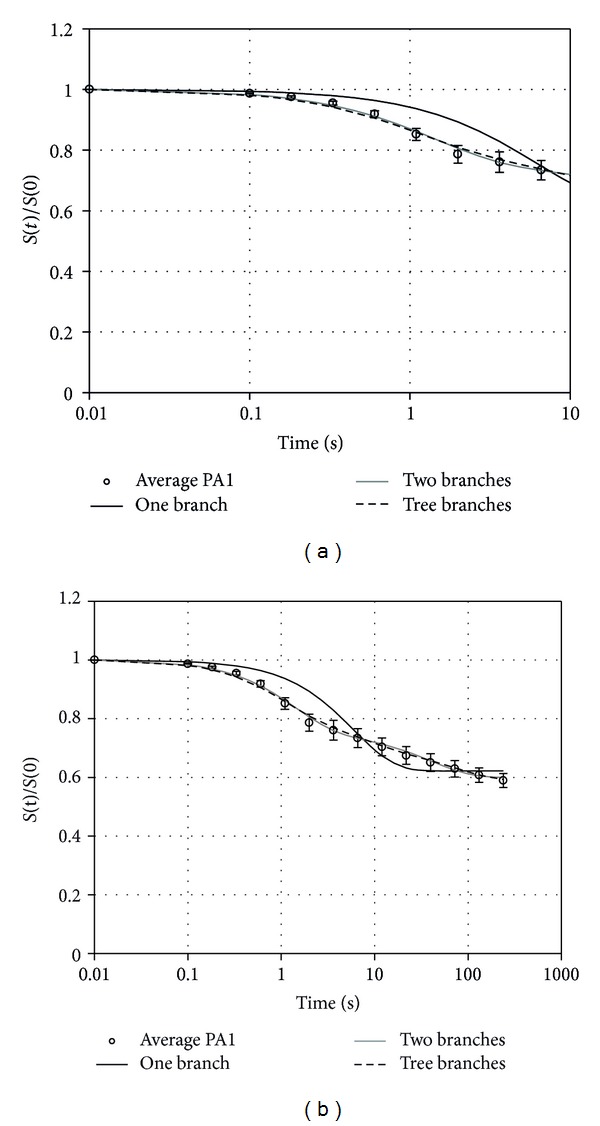
Average experimental data (open circles) and SD for the samples taken from the plantar aponeurosis of one of the donors, as normalized stress versus time. The numerical solutions refer to a viscoelastic model with one, two, and three viscous branches. The stress relaxation is reported up to 10 s (a) and for the whole relaxation process (b).

The comparison between average experimental data originating from the samples of one of the donors and numerical results obtained by fitting the constitutive model with different number of viscoelastic branches is shown in Figure 3. Average data are drawn from all the different stress relaxation tests and all the samples of the plantar aponeurosis. The constitutive model fitting was obtained by assuming one, two, three, and four viscoelastic branches, respectively. The curves of the stress relaxation tests are represented in Figure 3(a) up to time 10 s versus a logarithmic time scale in order to allow for a better view of the fitting in the first time instants.  Figure 3(b) shows the same curves up to time 240 s. The data fitting computed by means of a model with four viscoelastic branches appeared superimposed to the curve obtained from the model with three viscous branches and, for this reason, it is not reported in the graphs. The values of relative stiffness and relaxation time of the different viscous branches are shown in Table 2.

**Figure 4:**
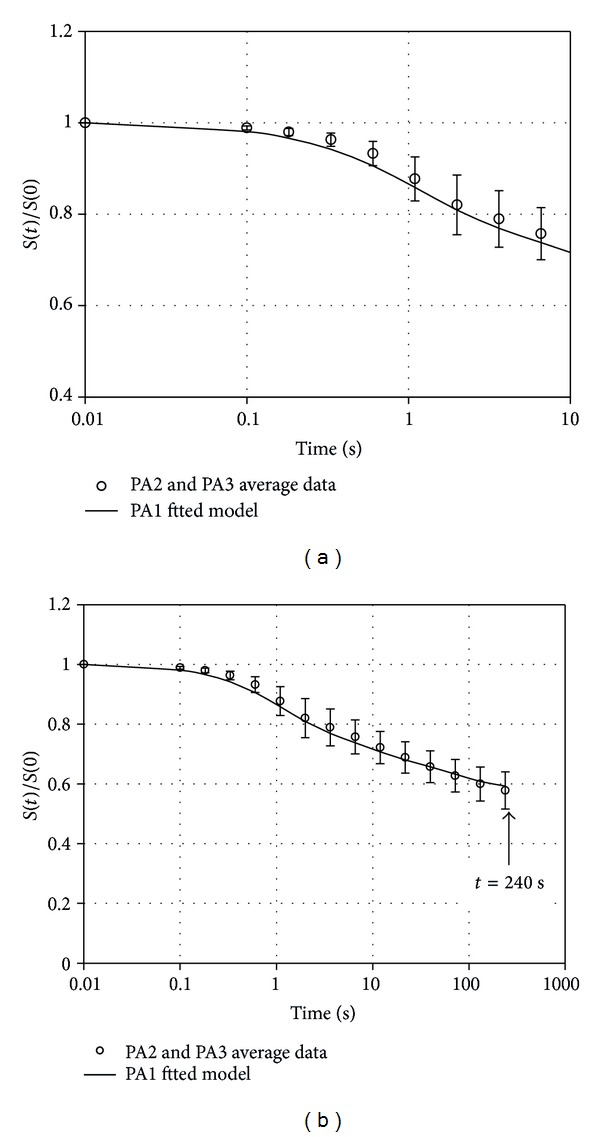
Comparison of numerical results (continuous line) and experimental data (open circles) for the stress relaxation process. Data are reported versus time up to the 10 s (a) and 240 s (b). The numerical solution is obtained by fitting the model on the samples taken from one of the donors. The experimental data in the chart refer to samples of the other two donors.

A comparison between numerical results obtained with the previous fitting with 3 viscous branches and experimental data of all the samples taken from the other two donors and for all the three levels of strain applied is shown in Figure 4. Also in this case data are reported versus a logarithmic time scale up to 10 s (Figure 4(a)) and 240 s (Figure 4(b)). The average residual stress after 240 s, expressed as a fraction of the beginning stress value, is 57.9% ± 6.2%.

**Figure 5:**
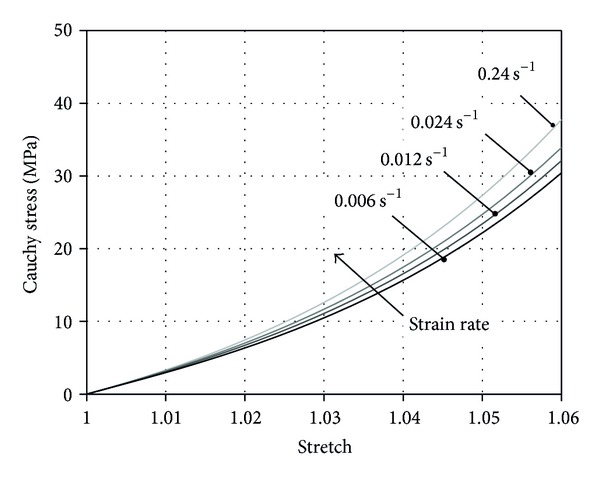
Cauchy stress versus stretch for a tensile ramp applied at different strain rates and obtained by numerical integration.

Finally, Figure 5 shows the stress-stretch response corresponding to a tensile ramp up to nominal strain of 6% developed at different constant strain rates (0.006 s^−1^, 0.012 s^−1^, 0.024 s^−1^, and 0.24 s^−1^). The curves are obtained by means of a time-integration scheme applied to (7). The hyperelastic function (4) is assumed with constitutive parameters of *μ* = 14.449 MPa, *k* = 254.02 MPa, and *α* = 10.397. These constitutive parameters are obtained by a fitting procedure of the hyperelastic model to experimental data of the plantar aponeurosis elastic response, taken from the literature [5, 13].

## 4. Discussions

The statistical analysis (ANOVA) performed among the stress relaxation processes for the different levels of strain applied did not show statistical difference, allowing us to reasonably suppose that stress relaxation percentage is not influenced by the strain applied, within the strain range investigated. This fact justifies the choice of a constitutive model based on the hypothesis of quasilinear viscoelasticity.

The choice of a constitutive model with three viscoelastic branches improves data fitting, mainly in the first time instants, if compared to a model with two viscoelastic branches. The numerical solution obtained from a model with four viscoelastic branches shows an increase of parameters number not justified in front of a marginal improvement of accuracy. Relaxation times for the model with three viscous processes show the presence of a short-, medium-, and long-term contribution spanning the order of magnitude of about 1 s, 10 s and 100 s, as a feature typically found in other soft tissues [18, 19, 26].

The model fitting is performed considering the average data taken from one donor and compared with data from the other two subjects for all the three levels of strain applied in the stress relaxation tests. Results reported confirm the suitability of the proposed model to describe the viscoelastic response. In fact, it is possible to observe that the model well fits both average data from tests at different levels of strain applied (Figure 2) and global average data from the two different plantar aponeurosis mentioned above (Figure 4). The good correlation between numerical results and experimental data is found in the entire relaxation process. The stress relaxation phenomena were monitored in a time interval up to 240 s at which end a residual stress of about 60% of the peak stress was found. Considering the stress decay rate, as deduced from the experimental data and in agreement with the corresponding numerical results, it is possible to observe that the viscous process can be considered almost completely developed, with stress decay rate below 0.007% s^−1^.

The comparison of the stress-stretch behaviour at different strain rates shown in Figure 5 allows for evaluating the possible effects on the stiffness due to a different rate of loading. The elongation corresponding to a nominal strain of 6%, a level included in the physiological range as reported in the literature, is obtained with the numerical integration in a time ranging from 0.25 to 10 s. As expected, according it viscoelastic properties, the plantar aponeurosis tissue exhibits an increasing stiffness concurrently with the strain rate increase. As first approximation, evaluating the stiffness of the tissue at 6% of strain by a secant modulus a difference is found close to 20% between the stiffness corresponding to the strain rate of 0.006 s^−1^ and the stiffness corresponding to the strain rate of 0.24 s^−1^.

The strain rate in the application of the elongation ramp preceding the stress relaxation phase was chosen to obtain an almost elastic response of the tissue but avoiding, at the same time, effects due to inertial forces. With the strain rate of 120%/s the developing of viscous phenomena in the ramp can be considered negligible. To evaluate this, the stress response for the effective strain history of a typical test at 8% of elongation was numerically estimated by applying a time-integration algorithm. The stress drop at the end of the ramp between the case of an ideal instantaneous elongation and the case of the effective elongation, which takes place in 0.067 s, was below 0.85%.

The choice of investigating the behavior of the tissue up to maximum nominal strain of 8% was made considering the physiological strain ranges generally accepted in the literature [6]. Uniaxial tensile tests were developed on few samples at this maximum level of strain, verifying the superposition of stress-strain cycles. This allows us to exclude damage phenomena in the tissue.

The limits of the present work must be considered in order to evaluate its significance and to plan further investigation on the mechanics of the plantar aponeurosis.

The analysis of the viscoelastic behavior of the plantar aponeurosis along the medial-lateral direction could require distinguishing between viscous behavior of ground matrix and fibers that in this work are not considered. On the other hand, it must be noted that the mechanical function of the plantar aponeurosis in supporting the foot arch is mainly along the proximal-distal direction and, as consequence, mainly related to the mechanical properties of the fibers aligned to this axis. The investigation of the mechanical properties in the medial-lateral direction could be helpful in clarifying the role of interaction between the ground matrix and fibers in the mechanical behavior of the tissue.

It will be important to extend the investigation to a greater number of subjects in order to achieve more exhaustive database. On the other hand, the literature concerning experimental testing of plantar aponeurosis is not as evolved and shows how the investigation suffers because of a general limitation of the samples at disposal [5, 8]. This confirms the difficulty in obtaining a large number of samples.

An issue that could be considered in order to reach a more exhaustive description of this phenomenon is represented by investigating differences between fresh and frozen tissues and to exclude that the independency of the stress relaxation from the level of strain can be related to a modification of the frozen tissue. However, it was shown that the frozen process should not play a damaging role [29–31]. The study could comprise the evaluation of the viscoelastic phenomena in plantar aponeurosis of younger subjects and, if need be, how these phenomena could differ from those reported in the present work where donors were over 65 years of age. It is evident that the testing on fresh samples or taken from younger subjects appears much more difficult.

## 5. Conclusion

The investigation is mainly addressed to viscous phenomena of the plantar aponeurosis according to different samples and placement of the samples. The present analysis suffers because of the limited number of donors considered. In any case, the comparison of experimental data taken from stress relaxation tests and numerical results shows a good capability of the constitutive model to describe the stress relaxation phenomena of the plantar aponeurosis tissue in a strain range that can be referred to physiological conditions, according to the literature. A valid compromise between complexity of the model, due to the number of the constitutive parameters, and capability to well describe the viscoelastic phenomena is found assuming a model with three viscous branches.

The main purpose of defining this constitutive model for the plantar aponeurosis is the adoption of this model for investigating foot biomechanics using a numerical approach. The capability of the constitutive model to include the viscoelastic response of the plantar aponeurosis tissue represents an improvement with respect to the usually defined models in the literature. Indeed, the proposed model is defined according to experimental data that refer to specific regions of the plantar aponeurosis (proximal, central, and distal) showing a general homogeneity of the stress relaxation phenomena. This can be useful to investigate the role of the plantar aponeurosis in the deformation of the foot arch and induced effects on foot overall structure. Including time-dependent behavior of the plantar aponeurosis it is possible to model the biomechanics of the foot as a function of different rates of loading, as consequence offering an effective description of internal forces acting during gait.

The proposed model could be useful for investigating, from a biomechanical point of view, important pathologies. For example, it is known that in plantar fasciitis the patient feels the major pain after a period of resting, typically with the foot loading at the first steps in the morning. It is possible that the viscoelastic characteristics of the plantar aponeurosis play a role in this, affecting the stiffness of the tissue. Therefore, it is likely that a better understanding of such loading-related pathologies can be obtained thanks to the numerical approach presented in this work.

## Figures and Tables

**Table 1:** Mean and SD of stress relaxation at time instants of 0.1 s, 6 s, 72 s, and 240 s for the different relaxation tests.

	*t* = 0.1 s	*t* = 6 s	*t* = 72 s	*t* = 240 s
*ε* = 4%	0.90 (0.07)	0.73 (0.06)	0.61 (0.06)	0.57 (0.07)
*ε* = 6%	0.91 (0.06)	0.76 (0.05)	0.63 (0.04)	0.58 (0.05)
*ε* = 8%	0.92 (0.06)	0.77 (0.04)	0.64 (0.03)	0.60 (0.04)

**Table 2:** Viscous constitutive parameters obtained adopting a model with one, two, three, and four viscous branches, respectively.

#	*γ*_1_	*τ*_1_ (s)	*γ*_2_	*τ*_2_ (s)	*γ*_3_	*τ*_3_ (s)	*γ*_4_	*τ*_4_ (s)
1	0.39	6.0						
2	0.25	1.40	0.15	39.87	—	—	—	—
3	0.19	1.00	0.11	7.02	0.12	75.21	—	—
4	0.03	0.10	0.21	1.76	0.11	23.87	0.10	292.88
